# Magnesium Administration as a Treatment for Perioperative Atrial Fibrillation following Cardiac Surgery: Safety and Efficacy

**DOI:** 10.5761/atcs.oa.26-00129

**Published:** 2026-10-22

**Authors:** Takafumi Terada, Yoshiyuki Tokuda, Yoshimori Araki, Akihiro Kobayashi, Osamu Kawaguchi, Eriko Koike, Masato Mutsuga

**Affiliations:** 1 Department of Cardiac Surgery, Nagoya University Graduate School of Medicine, Nagoya, Aichi, Japan; 2 Department of Cardiac Surgery, JA Aichi Koseiren Toyota Kosei Hospital, Toyota, Aichi, Japan

**Keywords:** perioperative atrial fibrillation, magnesium therapy

## Abstract

**Purpose:**

Atrial fibrillation (AF) is a common complication after cardiac surgery, impacting recovery and outcomes. Magnesium has electrophysiological and rate-controlling effects in AF. This study evaluated the safety and the temporal association between therapeutic intravenous magnesium administration and rhythm outcomes in patients with postoperative atrial fibrillation (POAF) after cardiac surgery.

**Methods:**

Between January 2009 and December 2016, 714 patients undergoing cardiac surgery were evaluated. After excluding 49 patients with a history of AF, 130 developed new-onset POAF. Of these, 111 patients (85.4%) received intravenous magnesium according to our institutional protocol, whereas 19 patients (14.6%) did not. Magnesium (20 mEq per dose, 1–3 doses) was administered with other antiarrhythmic agents in 40 patients (36.0%) and as monotherapy in 71 patients (64.0%). The principal efficacy analyses were restricted to the magnesium-monotherapy cohort. Serum magnesium levels after administration were monitored in 35 patients.

**Results:**

Among the 71 patients treated with magnesium monotherapy, the heart rate significantly decreased from 114 ± 32.3 bpm to 100 ± 39.7 bpm (P <0.01), with no significant change in blood pressure. Sinus rhythm conversion occurred in 34 patients (47.9%) within 3 hours and in 60 patients (84.5%) within 24 hours. In multivariable logistic regression analysis, a higher preoperative serum magnesium concentration (adjusted OR, 1.31 per 0.1 mg/dL increase; 95% profile-likelihood CI, 1.07–1.65; P = 0.014) and later postoperative onset of POAF (adjusted OR, 1.32 per postoperative day; 95% CI, 1.07–1.68; P = 0.015) were associated with 3-hour conversion. No serious adverse events attributable to magnesium were observed in the monotherapy cohort. However, because no untreated-comparator group was available, spontaneous conversion cannot be distinguished from a treatment effect.

**Conclusion:**

Intravenous magnesium monotherapy was associated with heart rate reduction without significant blood pressure deterioration and was followed by early sinus rhythm conversion in a substantial proportion of patients with POAF. These observational findings support the feasibility and apparent safety of this approach, but do not establish a causal antiarrhythmic effect. Prospective controlled studies are required.

## Abbreviations

AF
: atrial fibrillation
IV
: intravenous
Mg
: magnesium
POAF
: postoperative atrial fibrillation

## Introduction

Atrial fibrillation (AF) is a common complication of cardiac surgery. Among cardiac surgery patients, its incidence rate is 25%–50%, and it commonly occurs 2–4 days after surgery.^1,2)^ Postoperative atrial fibrillation (POAF) causes hemodynamic instability; prolonged intensive care unit (ICU) and hospital stays; thromboembolic complications, such as cerebral infarction; increased hospital expenses; and worse short- and long-term outcomes.^3)^ Several clinical factors, including older age, a larger left atrial diameter, reduced left ventricular ejection fraction, heart failure, chronic obstructive pulmonary disease, hypertension, and prior myocardial infarction, have been associated with an increased risk of POAF.^4)^

Some treatments for POAF, such as amiodarone or beta-blockers, are recommended; however, these recommendations are mainly based on evidence from prophylactic trials.^5–10)^

Intravenous magnesium supplementation is widely used to prevent or treat arrhythmias after cardiac surgery.^11)^ The effectiveness of prophylactic intravenous magnesium supplementation has been reported in previous studies,^12,13)^ and magnesium supplementation is recommended when the serum magnesium level is low or total-body magnesium depletion is suspected.^5)^ On the other hand, although the efficacy of intravenous magnesium therapy for common AF has been reported, evidence regarding its therapeutic use for POAF after cardiac surgery remains limited.^14,15)^

We have focused on the therapeutic role of IV magnesium in POAF and have routinely administered IV magnesium sulfate to patients who developed POAF according to our protocol.

Existing guidelines do not strongly recommend magnesium administration for the treatment of POAF; therefore, we retrospectively evaluated the efficacy and safety of the therapeutic intravenous magnesium administration protocol used in our hospital.

## Materials and Methods

### Inclusion and exclusion criteria

From January 2009 to December 2016, 714 adult patients underwent cardiac surgery, and 179 developed AF after open-heart surgery. Forty-nine patients had a preoperative history of AF and were excluded from the new-onset POAF cohort. Among the remaining 130 patients with new-onset POAF, 111 received intravenous magnesium and constituted the principal study cohort; 19 did not receive magnesium. The clinical course of these 19 patients was reviewed descriptively in response to the heterogeneity of treatment selection, but they were not considered an untreated control group because treatment allocation was not randomized and subsequent management was heterogeneous (**Fig. 1**). We reviewed the medical records of all included patients.

**Fig. 1:**
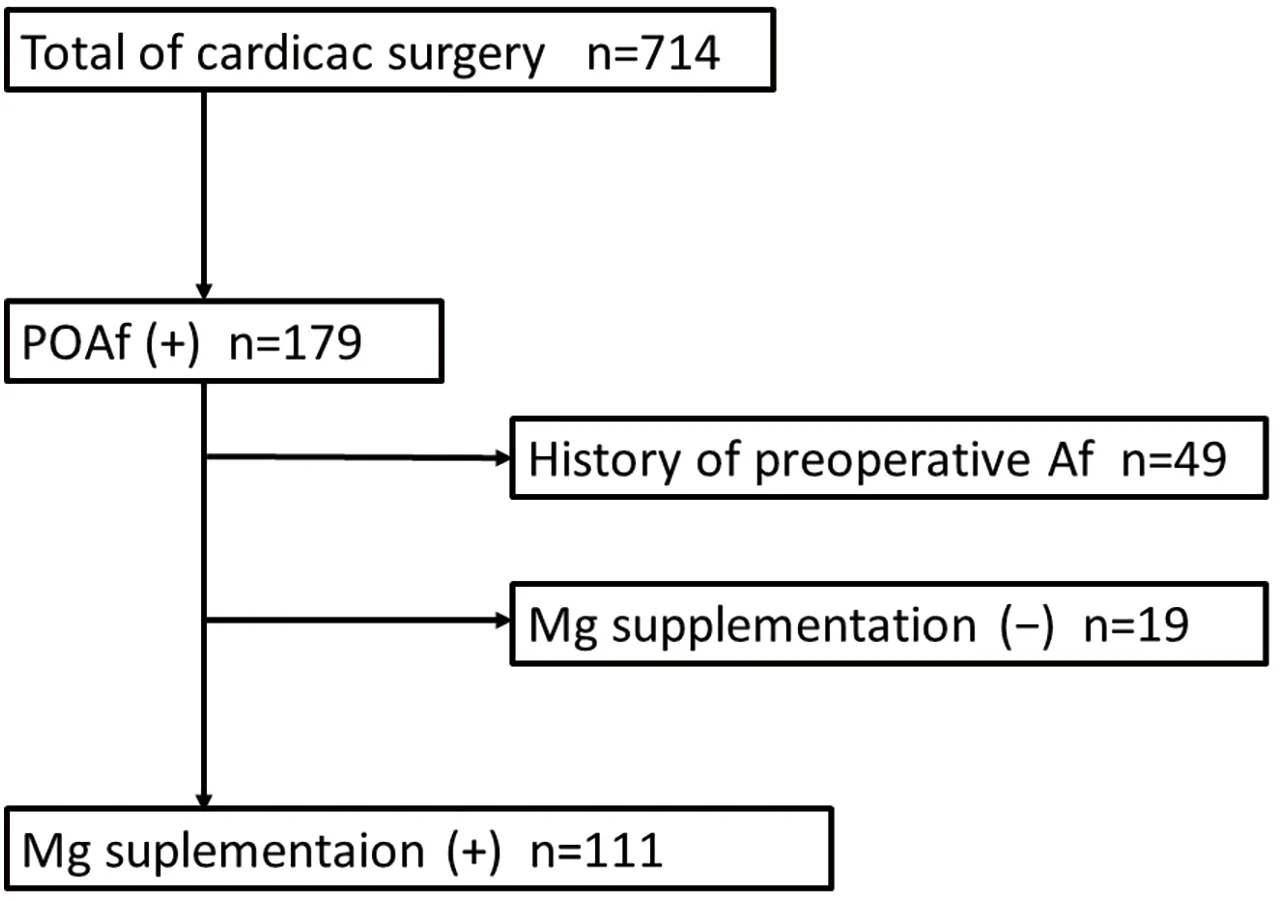
Inclusion criteria.

### Treatment protocol and data collection

Once POAF was diagnosed, magnesium sulfate (20 mEq/100 mL saline) was administered intravenously over 1 hour according to our institutional treatment protocol. The same dose could be repeated once daily for up to 3 days according to the treating physician’s assessment. In principle, treatment was initiated without waiting for a serum magnesium measurement, and magnesium was not administered prophylactically for POAF. There was no standardized institutional algorithm for adding other antiarrhythmic drugs; the choice and timing of additional treatment were determined by the treating physician according to the patient’s clinical condition.

The patients’ clinical data included demographic characteristics, the surgical procedure, the postoperative day and location of AF onset, the use of beta-blockers preoperatively and at AF onset, catecholamine use, cardiopulmonary bypass (CPB) use, preoperative renal function, perioperative electrolyte levels, sinus rhythm conversion, vital signs before and after magnesium administration, hospital length of stay, and complications. The principal efficacy analyses were restricted to the 71 patients treated with magnesium monotherapy to minimize confounding by concomitant antiarrhythmic treatment.

The primary efficacy endpoint was early sinus rhythm conversion within 3 hours after initiation of intravenous magnesium. This time point was selected to assess an early treatment response after the 1-hour infusion and to reduce the increasing influence of spontaneous conversion and subsequent therapeutic interventions over longer observation periods. Patients were considered to have achieved successful sustained conversion only when sinus rhythm was restored within the specified interval without subsequent recurrence during the observation period. A sensitivity analysis using conversion within 6 hours was also performed. A 12-hour multivariable sensitivity analysis was not performed because the sample size and number of outcome events were considered insufficient for additional reliable multivariable modeling. Serum magnesium levels after treatment were assessed according to the amount of intravenous magnesium administered and the preoperative estimated glomerular filtration rate.

### Statistical analysis

Continuous variables were compared using Student’s t-test, Welch’s t-test, or the Mann–Whitney U test, as appropriate, and categorical variables were compared using the χ² test or the 2-sided Fisher’s exact test. Multivariable logistic regression was performed in the magnesium-monotherapy cohort to evaluate factors independently associated with sinus rhythm conversion within 3 hours. The model included the preoperative serum magnesium concentration (scaled per 0.1 mg/dL), the postoperative day of POAF onset, age, catecholamine use at POAF onset, CPB use, preoperative estimated glomerular filtration rate (eGFR), and valve-related surgery. Odds ratios (ORs) and 95% profile-likelihood confidence intervals (CIs) were calculated. Variance inflation factors were assessed for multicollinearity. Hospital length of stay was compared according to 3-hour conversion status using the Mann–Whitney U test. Differences were considered statistically significant at P <0.05. Statistical analyses were performed using EZR (Saitama Medical Center, Jichi Medical University, Saitama, Japan).^16)^

## Results

As shown in **Table 1**, 79 patients (71.2%) were male, and the mean age at surgery was 70 ± 10.5 years. AF occurred at a mean of 3.1 ± 2.6 postoperative days, and 55 patients (49.5%) developed AF in the ICU. Of the 111 patients treated with intravenous magnesium, 71 (64.0%) received magnesium monotherapy and 40 (36.0%) received magnesium with additional antiarrhythmic medication. Forty patients (36.0%) had been receiving beta-blockers before surgery, whereas 42 patients (37.8%) were receiving beta-blockers at POAF onset. Additional drugs used with magnesium included beta-blockers in 17 patients (intravenous in one), Vaughan Williams class I antiarrhythmic agents in 17 patients (class Ia: cibenzoline succinate, n = 5; disopyramide, n = 1; class Ic: pilsicainide, n = 13), amiodarone in 6, non-dihydropyridine calcium-channel blockers in 4, and digitalis in 3; some patients received more than 1 agent. The mean duration of intravenous magnesium supplementation was 2.4 ± 0.8 days.

**Table 1:** Patients’ characteristics

Age at operation, years	70 ± 10.5	
Sex, male (%)	79 (71.2%)	
Length of hospital stay, days	34.6 ± 40.0	
Length of ICU stay, days	2.8 ± 2.5	
AF onset, postoperative days	3.1 ± 2.6	
Number of patients in ICU at AF onset (%)	55 (49.5%)	
C.A.-dependent at AF onset	17 (15.3%)	
Surgical procedure	CPB use	84 (CABG, 24; valve, 21; aorta, 19; other, 20)
	Off-pump CABG	27
Preoperative beta-blocker use (%)	40 (36.0%)	
Beta-blocker use at onset of AF (%)	42 (37.8%)	

ICU, intensive care unit; AF, atrial fibrillation; CPB, cardiopulmonary bypass; CABG, coronary artery bypass graft; C.A., catecholamine

Among the 19 patients with new-onset POAF who did not receive intravenous magnesium, 7 received no additional pharmacological treatment. Class I antiarrhythmic drugs were used in 6 patients, amiodarone in 1, beta-blockers in 4, and non-dihydropyridine calcium-channel blockers in 2; some patients received more than 1 medication. Six patients converted to sinus rhythm within 24 hours, including 3 within 3 hours. One patient underwent electrical cardioversion, 1 subsequently underwent catheter ablation, and 2 were discharged with persistent arrhythmia. Because treatment selection was nonrandomized and management was heterogeneous, these patients were not used as a comparator group.

Among the 71 patients treated with magnesium monotherapy, sinus rhythm conversion occurred in 34 (47.9%), 43 (60.6%), 52 (73.2%), and 60 (84.5%) patients within 3, 6, 12, and 24 hours, respectively (**Fig. 2**). **Figure 2** presents the cumulative probability of sinus rhythm conversion (1 − Kaplan–Meier estimate), with 95% Cls, censoring information, and numbers at risk.

**Fig. 2:**
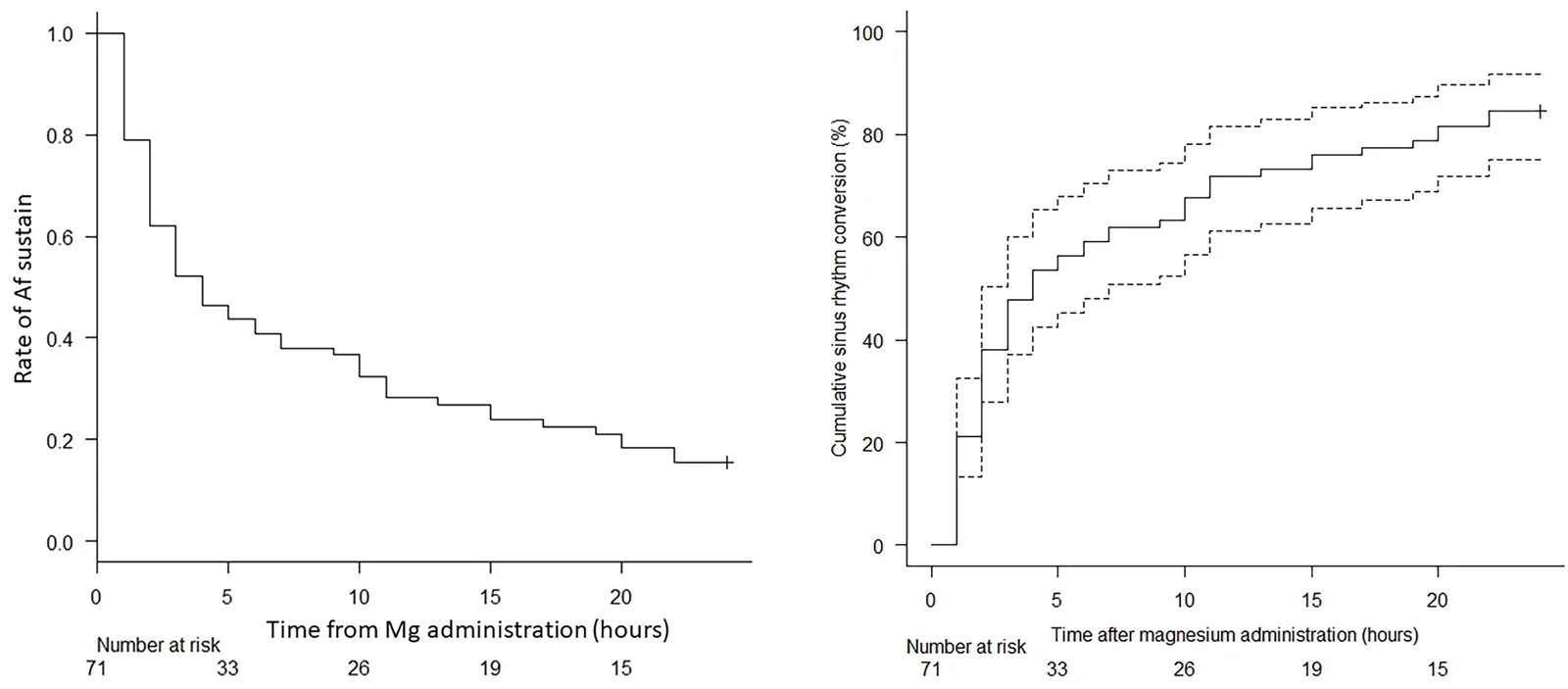
Cumulative sinus rhythm conversion after intravenous magnesium administration. The curve represents the cumulative probability of sinus rhythm conversion after intravenous magnesium administration in the magnesium-monotherapy cohort. Sinus rhythm conversion was treated as the event. Patients who did not achieve sinus rhythm conversion during the 24-hour observation period were censored at 24 hours. Dashed lines indicate 95% confidence intervals, censoring is indicated by tick marks, and numbers at risk are shown below the x-axis.

The patients’ hemodynamic status before and after magnesium administration is shown in **Fig. 3**. Although blood pressure did not change significantly after magnesium administration, the heart rate decreased significantly from 114 ± 32.3 bpm to 100 ± 39.7 bpm (P <0.01).

**Fig. 3:**
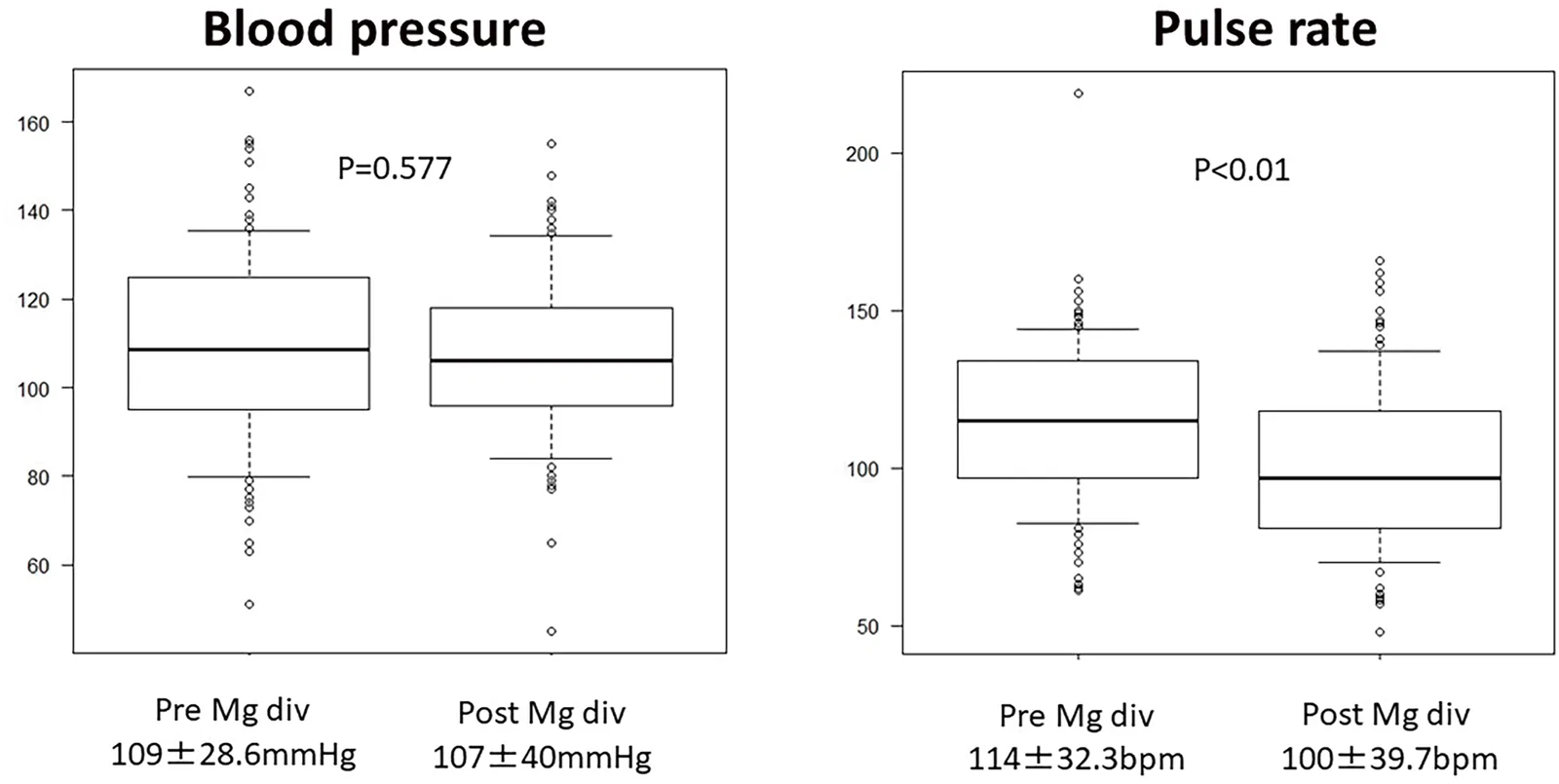
Hemodynamic status before and after magnesium administration.

The 34 patients in whom AF converted to sinus rhythm within 3 hours after magnesium administration were compared with patients in whom AF persisted for ≥3 hours. In the univariate analysis, the preoperative serum magnesium concentration was higher in the early-conversion group (**Table 2**). In the additional multivariable logistic regression analysis (68 complete cases), a higher preoperative serum magnesium concentration remained associated with 3-hour conversion (adjusted OR, 1.31 per 0.1 mg/dL increase; 95% profile-likelihood CI, 1.07–1.65; P = 0.014), as did later postoperative onset of POAF (adjusted OR, 1.32 per postoperative day; 95% CI, 1.07–1.68; P = 0.015). Age, catecholamine use at AF onset, CPB use, preoperative eGFR, and valve-related surgery were not significantly associated with early conversion. Variance inflation factors were 1.15–1.44, indicating no important multicollinearity (**Table 3**). Beta-blocker use was not significantly associated with 3-hour conversion in the unadjusted analyses (**Table 4**).

**Table 2:** Efficacy of magnesium administration and patients’ characteristics

	SR conversion within 3 hour, n = 34	AF persisted for >3 hour, n = 39	P value
Male	25 (73.5%)	28 (71.8%)	0.694
Age	69 ± 9.8	70 ± 12	0.799
Systolic BP (mmHg)	109 ± 22	110 ± 16	0.87
HR (bpm)	113 ± 26	109 ± 23	0.545
C.A.-dependent at AF onset	5 (14.7%)	4 (10.3%)	0.826
AF onset (postoperative day)	3.8 ± 2.4	4.6 ± 3.3	0.217
Hypokalemia (K <4.0 mmol/L)	5 (14.7%)	8 (20.5%)	0.734
Serum K (mmol/L) at AF onset	4.37 ± 0.42	4.26 ± 0.37	0.283
Use of CPB	24 (70.6%)	33 (84.6%)	0.245
Preoperative eGFR (mL/min/1.73 m²)	60.8 ± 24.4	63.1 ± 24.3	0.694
Preoperative serum Mg (mg/dL)	2.19 ± 0.38	2.03 ± 0.24	0.034
Low EF <40%	3 (8.8%)	4 (10.3)	1

SR, sinus rhythm; AF, atrial fibrillation; BP, blood pressure; HR, heart rate; C.A., catecholamine; CPB, cardiopulmonary bypass; eGFR, estimated glomerular filtration rate; Mg, magnesium; EF, ejection fraction

**Table 3:** Multivariable logistic regression analysis of factors associated with successful sinus rhythm conversion within 3 hours after intravenous magnesium administration

Variable	Adjusted OR	95% CI*	P value
Preoperative serum Mg, per 0.1 mg/dL increase	1.31	1.07–1.65	0.014
Postoperative day of AF onset, per 1-day increase	1.32	1.07–1.68	0.015
Age, per 1-year increase	0.98	0.93–1.03	0.480
C.A.-dependent at AF onset	3.92	0.71–26.10	0.128
Use of CPB	0.50	0.11–2.06	0.346
Preoperative eGFR, per 1 mL/min/1.73 m² increase	1.00	0.98–1.03	0.902
Valve procedure	0.58	0.15–2.05	0.405

*Values are adjusted ORs with 95% profile-likelihood CIs. P values were obtained using Wald tests. A valve procedure was defined as a surgical procedure that included valve surgery. ORs for continuous variables represent the change associated with the increment specified for each variable. The multivariable analysis included 68 patients with complete data.

Mg, magnesium; AF, atrial fibrillation; C.A., catecholamine; CPB, cardiopulmonary bypass; eGFR, estimated glomerular filtration rate; ORs, odds ratios; CIs, confidence intervals

**Table 4:** Influence of beta-blocker usage preoperatively and at the onset of AF

Preoperative use of β-blocker	Use (n = 36)	No use (n = 42)	P value
SR conversion within 3 hour	15 (41.7%)	19 (45.2%)	0.93
Use of β-blocker at POAF onset	Use (n = 38)	No use (n = 38)	P value
SR conversion within 3 hour	16 (42.1%)	18 (47.4%)	0.81

SR, sinus rhythm; AF, atrial fibrillation; POAF, postoperative atrial fibrillation

In the 6-hour sensitivity analysis, the association between the preoperative serum magnesium concentration and conversion was attenuated and was not statistically significant (OR, 1.09 per 0.1 mg/dL increase; 95% profile-likelihood CI, 0.93–1.31; P = 0.315), suggesting that predictors of very early conversion may differ from those of later conversion.

Baseline and peri-POAF characteristics were also compared between the magnesium-monotherapy and combination-therapy groups (**Supplementary Table 1**). Beta-blocker use at POAF onset was more frequent in the monotherapy group (49.3% vs. 17.5%, P = 0.001), the heart rate before magnesium administration was lower (median, 112 vs. 126 bpm, P = 0.018), and the preoperative serum magnesium concentration was slightly lower (median, 2.10 vs. 2.15 mg/dL, P = 0.018). Other evaluated characteristics, including age, sex, postoperative day and ICU location of AF onset, catecholamine use, serum potassium level, systolic blood pressure, renal function, CPB use, valve-related surgery, and preoperative beta-blocker use, did not differ significantly between the groups. These differences indicate potential treatment-selection bias.

Hospital length of stay did not differ significantly according to early conversion status: the median was 25 days (IQR, 23–29.5) in patients with 3-hour conversion and 27 days (IQR, 23–29) in those without early conversion (P = 0.560). ICU length of stay was not analyzed as an outcome of conversion because approximately half of the POAF episodes occurred after transfer to the general ward. No stroke occurred in the study cohort. Recurrent AF was not analyzed as an independent secondary outcome because the absence of subsequent recurrence was incorporated into the definition of successful sustained conversion.

With regard to safety, ventricular tachycardia occurred in 1 patient after magnesium administration; however, pilsicainide had been administered before the onset of ventricular tachycardia. No serious adverse events attributable to magnesium were observed in the magnesium-monotherapy cohort. Serum magnesium levels were monitored during the latter phase of the study (n = 35); the mean serum magnesium concentration after administration was 2.59 ± 0.49 mg/dL. As shown in **Fig. 4**, there was no significant relationship between the increase in serum magnesium concentration and either the amount of magnesium administered or the preoperative eGFR. The increase in serum magnesium concentration after 3 doses remained below 1.5 mg/dL in all monitored patients, including those with impaired renal function, and no severe hypermagnesemia was observed. Because serum magnesium was generally not measured immediately before treatment, these observations do not establish the safety of empiric administration in all patients, particularly those at risk of magnesium accumulation.

**Fig. 4:**
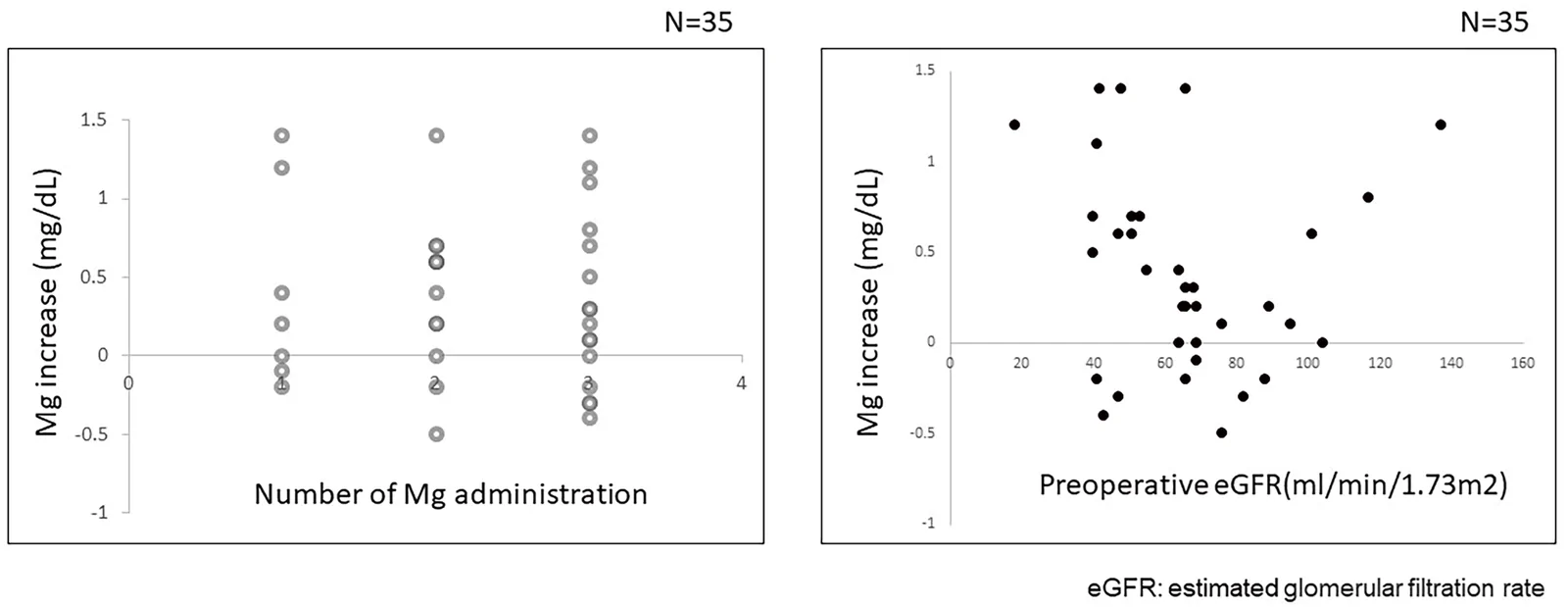
Increase in serum magnesium level according to the amount of magnesium administered and the estimated glomerular filtration rate.

## Discussion

Current guidelines for POAF recommend the prophylactic use of beta-blockers and, in selected settings, antiarrhythmic agents such as amiodarone, as well as magnesium supplementation when the serum magnesium level is low. However, no treatment is strongly recommended specifically for established POAF once it occurs.^5–10)^

In this study, we evaluated the safety and temporal rhythm response following therapeutic intravenous magnesium administration for POAF after cardiac surgery. In the magnesium-monotherapy cohort, 47.9%, 60.6%, 73.2%, and 84.5% of patients converted to sinus rhythm within 3, 6, 12, and 24 hours, respectively. The heart rate decreased significantly without a significant reduction in blood pressure, and no serious adverse events attributable to magnesium were observed in the monotherapy cohort. However, these conversion rates must be interpreted in the context of the natural history of POAF and cannot be attributed solely to magnesium in the absence of a comparator group.

POAF is frequently self-limiting. In a cardiac surgery cohort reported by Soucier et al., 131 of 163 patients (80%) managed without class I or III antiarrhythmic drugs converted to sinus rhythm within 24 hours.^17)^ Thus, a substantial proportion of the 84.5% 24-hour conversion rate observed in our cohort may represent natural recovery rather than a pharmacological effect of magnesium. This is particularly important because the present study had no untreated- or active-comparator group. Accordingly, our data demonstrate a temporal association between magnesium administration and subsequent rhythm conversion, but do not establish causal efficacy.

We selected 3 hours as the primary endpoint to characterize early conversion after the 1-hour magnesium infusion, when the temporal relationship with treatment was closest and before spontaneous recovery and subsequent interventions increasingly influenced rhythm status. The 3-hour threshold should not be interpreted as an established physiological cutoff. In the 6-hour sensitivity analysis, the association between the preoperative magnesium concentration and conversion was attenuated and was no longer statistically significant, supporting a cautious interpretation of the 3-hour predictor analysis. A 12-hour multivariable sensitivity analysis was not undertaken because the limited sample size and event distribution would make additional multivariable modeling unstable.

Because perioperative electrolyte disturbances, particularly hypokalemia, contribute to the development of POAF, many institutions monitor and maintain electrolyte concentrations within the physiological range after cardiac surgery. Magnesium increases the threshold for automaticity and prolongs atrial refractoriness. In addition, during hypokalemia, magnesium supplementation may facilitate renal potassium conservation by reducing potassium secretion through effects on renal potassium channels.^18,19)^

Regarding intravenous magnesium supplementation as prophylaxis, some guidelines recommend its use when the serum magnesium level is low.^5)^ This recommendation is partly based on reports showing a higher incidence of POAF in patients with lower serum magnesium concentrations, including the study by Zaman et al.^20)^. However, the effects of prophylactic magnesium supplementation remain controversial. Recent meta-analyses have reported that prophylactic intravenous magnesium supplementation may reduce the incidence of POAF after cardiac surgery.^12,21)^ Conversely, other meta-analyses have concluded that prophylactic magnesium supplementation does not prevent POAF after cardiac surgery.^22,23)^ These conflicting findings likely reflect heterogeneity among the included studies. Accordingly, routine magnesium supplementation for all patients undergoing cardiac surgery has not been established as standard therapy but remains a selective option.

Consistent with previous reports,^1,2)^ AF occurred at a mean of 3.1 postoperative days, and approximately half of the episodes occurred after transfer to the general ward. This setting is clinically relevant because continuous hemodynamic monitoring and immediate laboratory testing may be less readily available than in the ICU. In our institutional practice, magnesium was therefore generally administered promptly after recognition of POAF without waiting for a serum magnesium result. In the monotherapy cohort, the heart rate decreased without a significant change in blood pressure, and no serious adverse events attributable to magnesium were observed. These findings are reassuring regarding the feasibility in routine postoperative care, but they do not demonstrate that pre-treatment magnesium measurement is unnecessary or that empiric administration is universally safe.

From a pharmacokinetic perspective, Biesenbach et al. reported that a 20-mmol magnesium bolus after cardiac surgery produces a short-lived increase in serum magnesium concentration and that magnesium balance approaches neutrality within 12 hours.^24)^ In our series, the increase in serum magnesium concentration was not significantly related to the amount administered or the preoperative eGFR, and no severe hypermagnesemia was observed among the monitored patients. Nevertheless, serum magnesium was not measured at POAF onset or immediately before treatment. Therefore, we cannot determine whether the patients were magnesium deficient, whether treatment corrected an acute deficiency, or whether magnesium exerted an electrophysiological effect independent of deficiency. Future prospective studies should include serum magnesium measurements immediately before and after treatment.

A higher preoperative serum magnesium concentration was associated with early conversion in both univariate and adjusted analyses. Magnesium contributes to cardiac electrophysiology through its effects on ion transport, membrane stability, and atrial refractoriness. One possible hypothesis is that a higher preoperative serum magnesium concentration reflects better-preserved magnesium homeostasis or lower total-body magnesium depletion, which could influence the electrophysiological response to intravenous magnesium. However, this interpretation remains speculative because magnesium was not measured at POAF onset, and the serum concentration may not reflect intracellular magnesium stores. This finding should therefore be considered hypothesis-generating rather than evidence of a defined mechanism.

Magnesium is inexpensive and readily available, which may represent a practical advantage in clinical practice. However, the present study did not include a formal cost-effectiveness analysis, and the potential economic benefit of this approach requires dedicated evaluation.

Early sinus rhythm conversion did not translate into a measurable reduction in hospital length of stay in this cohort. ICU length of stay could not be meaningfully evaluated because approximately half of the POAF episodes developed after ICU discharge, and no strokes occurred. Readmission attributable specifically to recurrent AF was not systematically captured, and recurrence could not be evaluated as an independent endpoint because sustained conversion without subsequent recurrence formed part of the efficacy definition. Thus, this study was not designed or powered to demonstrate improvement in patient-centered outcomes, and larger prospective studies with predefined clinical endpoints are required.

### Limitations

This study has several important limitations. First, it was a retrospective, single-center observational study without an untreated or active-comparator group. Because POAF frequently converts spontaneously, particularly within the first 24–48 hours, the observed rhythm conversion cannot be causally attributed to magnesium. Second, treatment escalation and the addition of other antiarrhythmic drugs were physician-dependent, and baseline differences between the monotherapy and combination-therapy groups indicate potential treatment-selection bias. Third, the serum magnesium concentration was generally not measured at POAF onset or immediately before treatment; therefore, we cannot distinguish correction of magnesium deficiency from a pharmacological effect independent of deficiency. Fourth, the sample size and number of events limited the number of covariates that could be included in the multivariable analyses and precluded a reliable 12-hour multivariable sensitivity analysis. Finally, clinically meaningful outcomes such as ICU length of stay, stroke, recurrent AF as an independent endpoint, and AF-related readmission could not be fully evaluated, and early conversion was not associated with a shorter hospital stay. Prospective, controlled, multicenter studies with standardized treatment algorithms, peri-treatment magnesium measurements, and predefined patient-centered outcomes are required.

## Conclusion

In this retrospective cohort, intravenous magnesium monotherapy for POAF was associated with a significant reduction in heart rate without a significant decrease in blood pressure, and 47.9% of patients achieved sustained sinus rhythm conversion within 3 hours. A higher preoperative serum magnesium concentration and later postoperative onset of POAF were associated with early conversion after multivariable adjustment. No serious adverse events attributable to magnesium were observed in the monotherapy cohort. However, because spontaneous conversion is common and no comparator group was available, these findings do not establish causal antiarrhythmic efficacy. Prospective controlled studies are needed to determine the efficacy, mechanism, and clinical benefit of this simple treatment strategy.

## Supplementary Materials

Supplemental Table 1 Comparison of clinical characteristics between the combination-therapy and magnesium-monotherapy groups

## Declarations

### Data availability

The data that support the findings of this study are available from the corresponding author upon reasonable request.

## References

[1] LaPar DJ, Speir AM, Crosby IK, et al. Postoperative atrial fibrillation significantly increases mortality, hospital readmission, and hospital costs. Ann Thorac Surg 2014; 98: 527–33; discussion, 533. 10.1016/j.athoracsur.2014.03.039 25087786

[2] Rostagno C, La Meir M, Gelsomino S, et al. Atrial fibrillation after cardiac surgery: incidence, risk factors, and economic burden. J Cardiothorac Vasc Anesth 2010; 24: 952–8. 10.1053/j.jvca.2010.03.009 20570180

[3] Pillarisetti J, Patel A, Bommana S, et al. Atrial fibrillation following open heart surgery: long-term incidence and prognosis. J Interv Card Electrophysiol 2014; 39: 69–75. 10.1007/s10840-013-9830-6 24293173

[4] Yamashita K, Hu N, Ranjan R, et al. Clinical risk factors for postoperative atrial fibrillation among patients after cardiac surgery. Thorac Cardiovasc Surg 2019; 67: 107–16. 10.1055/s-0038-1667065 PMC6359984 30071562

[5] Frendl G, Sodickson AC, Chung MK, et al. 2014 AATS guidelines for the prevention and management of perioperative atrial fibrillation and flutter for thoracic surgical procedures. J Thorac Cardiovasc Surg 2014; 148: e153–93. 10.1016/j.jtcvs.2014.06.036 PMC4454633 25129609

[6] Joglar JA, Chung MK, Armbruster AL, et al. 2023 ACC/AHA/ACCP/HRS Guideline for the diagnosis and management of atrial fibrillation: a report of the American College of Cardiology/American Heart Association Joint Committee on Clinical Practice Guidelines. J Am Coll Cardiol 2024; 83: 109–279. 10.1016/j.jacc.2023.08.017 PMC11104284 38043043

[7] Filardo G, da Graca B, Sass DM, et al. Preoperative β-blockers as a coronary surgery quality metric: the lack of evidence of efficacy. Ann Thorac Surg 2020; 109: 1150–8. 10.1016/j.athoracsur.2019.07.056 31513778

[8] Arsenault KA, Yusuf AM, Crystal E, et al. Interventions for preventing post operative atrial fibrillation in patients undergoing heart surgery. Cochrane Database Syst Rev 2013; 2013: CD003611. 10.1002/14651858.CD003611.pub3 PMC7387225 23440790

[9] Brinkman W, Herbert MA, O’Brien S, et al. Preoperative beta-blocker use in coronary artery bypass grafting surgery: national database analysis. JAMA Intern Med 2014; 174: 1320–7. 10.1001/jamainternmed.2014.2356 24934977

[10] Kohsaka S, Miyata H, Motomura N, et al. Effects of preoperative beta blocker use on clinical outcomes after coronary artery bypass grafting: a report from the Japanese Cardiovascular Surgery Database. Anesthesiology 2016; 124: 45–55. 10.1097/ALN.0000000000000901 26517856

[11] Roscoe A, Ahmed AB. A survey of peri-operative use of magnesium sulphate in adult cardiac surgery in the UK. Anaesthesia 2003; 58: 363–5. 10.1046/j.1365-2044.2003.03082_1.x 12688270

[12] Miller S, Crystal E, Garfinkle M, et al. Effects of magnesium on atrial fibrillation after cardiac surgery: a meta-analysis. Heart 2005; 91: 618–23. 10.1136/hrt.2004.033811 PMC1768903 15831645

[13] Rostron A, Sanni A, Dunning J. Does magnesium prophylaxis reduce the incidence of atrial fibrillation following coronary bypass surgery? Interact Cardiovasc Thorac Surg 2005; 4: 52–8. 10.1510/icvts.2004.100339 17670355

[14] Ho KM, Sheridan DJ, Paterson T. Use of intravenous magnesium to treat acute onset atrial fibrillation: a meta-analysis. Heart 2007; 93: 1433–40. 10.1136/hrt.2006.111492 PMC2016911 17449500

[15] Davey MJ, Teubner D. A randomized controlled trial of magnesium sulfate, in addition to usual care, for rate control in atrial fibrillation. Ann Emerg Med 2005; 45: 347–53. 10.1016/j.annemergmed.2004.09.013 15795711

[16] Kanda Y. Investigation of the freely available easy-to-use software ‘EZR’ for medical statistics. Bone Marrow Transplant 2013; 48: 452–8. 10.1038/bmt.2012.244 PMC3590441 23208313

[17] Soucier RJ, Mirza S, Abordo MG, et al. Predictors of conversion of atrial fibrillation after cardiac operation in the absence of class I or III antiarrhythmic medications. Ann Thorac Surg 2001; 72: 694–7; discussion 697-8. 10.1016/s0003-4975(01)02817-x 11565643

[18] Raiten JM, Ghadimi K, Augoustides JG, et al. Atrial fibrillation after cardiac surgery: clinical update on mechanisms and prophylactic strategies. J Cardiothorac Vasc Anesth 2015; 29: 806–16. 10.1053/j.jvca.2015.01.001 26009291

[19] Polderman KH, Girbes AR. Severe electrolyte disorders following cardiac surgery: a prospective controlled observational study. Crit Care 2004; 8: R459–66. 10.1186/cc2973 PMC1065069 15566592

[20] Zaman AG, Alamgir F, Richens T, et al. The role of signal averaged P wave duration and serum magnesium as a combined predictor of atrial fibrillation after elective coronary artery bypass surgery. Heart 1997; 77: 527–31. 10.1136/hrt.77.6.527 PMC484795 9227296

[21] Fairley JL, Zhang L, Glassford NJ, et al. Magnesium status and magnesium therapy in cardiac surgery: a systematic review and meta-analysis focusing on arrhythmia prevention. J Crit Care 2017; 42: 69–77. 10.1016/j.jcrc.2017.05.038 28688240

[22] Cook RC, Yamashita MH, Kearns M, et al. Prophylactic magnesium does not prevent atrial fibrillation after cardiac surgery: a meta-analysis. Ann Thorac Surg 2013; 95: 533–41. 10.1016/j.athoracsur.2012.09.008 23141526

[23] Lancaster TS, Schill MR, Greenberg JW, et al. Potassium and magnesium supplementation do not protect against atrial fibrillation after cardiac operation: a time-matched analysis. Ann Thorac Surg 2016 Oct; 102(4): 1181–8. Epub 2016 Sep 2. PMID: 27596917; PMCID: PMC5030158. 10.1016/j.athoracsur.2016.06.066 PMC5030158 27596917

[24] Biesenbach P, Mårtensson J, Osawa E, et al. Magnesium supplementation: pharmacokinetics in cardiac surgery patients with normal renal function. J Crit Care 2018; 44: 419–23. 10.1016/j.jcrc.2018.01.011 29353118

