# Supplementary material for: Magnesium Administration as a Treatment for Perioperative Atrial Fibrillation following Cardiac Surgery: Safety and Efficacy
Source: Ann Thorac Cardiovasc Surg. 2026 Oct 22;32(1):26-00129. doi: 10.5761/atcs.oa.26-00129 (PMC13719229; doi:10.5761/atcs.oa.26-00129)
Supplement: Supplementary file 1 [file atcs-32-1-26-00129-s001.pdf]

Supplementary Table 1. Comparison of clinical characteristics between the combination-therapy and magnesium-monotherapy groups

Characteristic	Combination therapy (n=40)	Magnesium monotherapy (n=71)	P value
Age, years	71.5 (62.8–77.0)	72.0 (65.0–79.0)	0.487
Male sex, n (%)	27 (67.5)	52 (73.2)	0.522
Postoperative day of POAF onset	3 (3–4)	3 (2–5)	0.571
POAF onset in ICU, n (%)	20 (50.0)	35 (49.3)	1.000
Catecholamine use at POAF onset, n (%)	8 (20.0)	9 (12.7)	0.411
**Beta-blocker use at POAF onset, n (%)**	**7 (17.5)**	**35 (49.3)**	**0.001**
Serum potassium at POAF onset, mEq/L	4.3 (4.0–4.5)	4.4 (4.0–4.6)	0.511
**Heart rate before magnesium administration, beats/min**	**126 (110.5–140.0)**	**112 (94.8–127.5)**	**0.018**
Systolic blood pressure before magnesium administration, mmHg	109.5 (87.8–125.0)	108.0 (95.0–124.0)	0.613
**Preoperative serum magnesium, mg/dL**	**2.15 (2.10–2.40)**	**2.10 (1.90–2.30)**	**0.018**
Preoperative eGFR, mL/min/1.73 m²	66 (47–73.3)	59 (48–77)	0.871
Preoperative LVEF, %	61.6 (51.5–70.9)	62.9 (52.7–69.5)	0.970
Cardiopulmonary bypass use, n (%)	29 (72.5)	55 (77.5)	0.646
Valve-related procedure, n (%)	15 (37.5)	22 (31.0)	0.533
Preoperative beta-blocker use, n (%)	13 (32.5)	32 (45.1)	0.230

Data are presented as median (interquartile range) or n (%). Continuous variables were compared using the Mann–Whitney U test and categorical variables using Fisher's exact test.

Abbreviations: POAF, postoperative atrial fibrillation; ICU, intensive care unit; eGFR, estimated glomerular filtration rate; LVEF, left ventricular ejection fraction.
